# Disrupted neuregulin 1-ErbB4 signaling: Consequences of prenatal morphine exposure in rat pups and molecular gateway to neurological impairment

**DOI:** 10.1016/j.toxrep.2024.101687

**Published:** 2024-07-08

**Authors:** Samira Khayat, Hamed Fanaei, Hamid Hafezinouri, Abdolhakim Ghanbarzehi, Abolfazl Parsi-Moud, Ilia Mirzaei

**Affiliations:** a Pregnancy Health Research Center, Zahedan University of Medical Sciences, Zahedan, Iran; b Department of Midwifery, School of Nursing and Midwifery, Zahedan University of Medical Sciences, Zahedan, Iran; c Department of Physiology, School of Medicine, Zahedan University of Medical Sciences, Zahedan, Iran; d Laboratory Animal Research Center, Zahedan University of Medical Sciences, Zahedan, Iran; e Department of Neuroscience, School of Advanced Technologies in Medicine, Iran University of Medical Sciences, Tehran, Iran; f School of Medicine, Zahedan University of Medical Sciences, Zahedan, Iran

**Keywords:** Morphine, Neuregulin 1, ErbB4, Pregnancy, Inflammation, Neurodevelopment, BDNF

## Abstract

**Objective:**

Morphine exposure during pregnancy has detrimental effects on both the mother and her offspring, both during and after childbirth. This study aimed to investigate the impact of prenatal morphine exposure on rat pups and dams, specifically focusing on changes in Neuregulin-1 (Nrg-1)/ErbB4 gene expression, inflammation, and brain-derived neurotrophic factor (BDNF) levels.

**Materials and methods:**

Twenty female rats were randomized into two experimental groups:

1-Morphine Group: Dams received morphine throughout pregnancy. 2-Control Group: Dams received no interventions.

At the end of gestation, blood samples were collected from the dams. Subsequently, dams and their pups underwent tissue collection from the cortical area of the brain to evaluate the following parameters: Interleukin-6 (IL-6), Interleukin-10 (IL-10), total antioxidant capacity (TAC), Malondialdehyde (MDA), and Brain-derived neurotrophic factor (BDNF).

Additionally, RNA was extracted from the pup's cortical brain tissue for the assessment of gene expression levels of Neuregulin-1 (NRG-1) and ErbB-4 using quantitative real-time polymerase chain reaction (qrt-PCR).

**Results:**

The molecular investigation revealed a decrease in NRG-1 and ErbB-4 expressions in the brain cortex of offspring exposed to morphine during prenatal development. Additionally, the levels of IL-6 and IL-10 in both the serum and brain of both the mothers and their offspring in the morphine group were significantly higher compared to the control group. The morphine-exposed group also exhibited significantly lower levels of TAC and higher levels of MDA, indicating increased oxidative stress. Furthermore, the levels of BDNF in the morphine group were significantly lower compared to the control group.

**Conclusion:**

Prenatal morphine exposure in rats has detrimental effects on both the dams and their offspring. This study demonstrates that prenatal morphine exposure disrupts critical molecular pathways involved in neurodevelopment, inflammation, oxidative stress, and neurotrophic signaling. These findings suggest that prenatal morphine exposure can have long-lasting consequences for the offspring, potentially contributing to neurodevelopmental disorders and other health issues later in life.

## Highlights

• This study investigated the impact of prenatal morphine exposure on rat pups and dams.
• Prenatal morphine exposure decreases NRG-1 and ErbB-4 expression in the offspring's brain cortex.
• The morphine-exposed group had higher pro-inflammatory cytokine levels in both maternal serum and offspring brains.
• Prenatal morphine exposure reduced BDNF levels in the offspring brain cortex.
• Prenatal morphine exposure has lasting effects on offspring neurodevelopment and health.

## Graphical Abstract

**Figure:**
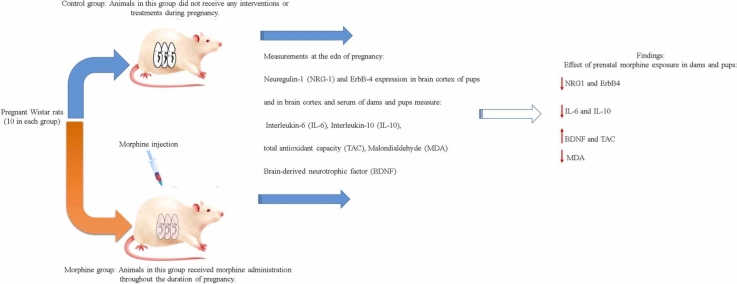

## 1 Introduction

Opioid dependence arises from repeated or continuous use of opioids. It involves a strong internal drive to use opioids, impaired ability to control use, and persistence of use despite harm or negative consequences (e.g., health problems, social issues) [1], [2], [3], [4].

Opioids can modify gene expression via transcriptional control and epigenetic changes, affecting specific genes like the mu opioid receptor (MOR) and potentially neuroplasticity-related genes [5]. They primarily act through mu (MOR), delta (DOR), and kappa (KOR) receptors, regulating neurotransmission by modulating release presynaptically and neuron activity postsynaptically [5], [6]. Opioids also possess immunomodulatory effects, influencing natural killer cell function, T and B cell responses, cytokine production, among others [7]. Further investigation is required to comprehensively grasp these intricate processes.

Morphine consumption during pregnancy causes complications for both the pregnant woman and her offspring [8]. Opiates have the ability to cross the placenta, blood-brain barrier, and breast milk, potentially leading to neurodevelopmental disorders in offspring [9], [10], [11] Opioid receptors are widely distributed throughout the brain, and their activation modulates various brain processes. In pregnant women who abuse opioids, opioid receptor stimulation can disrupt normal maternal behaviors necessary for infant care [10], [12], [13]. This disruption may contribute to complications such as premature rupture of membranes, meconium, and fetal respiratory distress, commonly observed in pregnancies affected by opioid abuse [12], [14].

Complications of opioid use by mothers may extend well into childhood and adulthood, and includes: inattention, hyperactivity, violence, and impulsive behaviors. [15], [16] Current evidence suggests that opioids alter the nervous system development, the mechanism of which is not well understood. [17] Research has demonstrated that opium use during pregnancy can adversely affect maternal behavior and fetal development. This is primarily attributed to the ability of opioids to alter gene expression, immune system processes, and neurotransmitter release [10], [12], [18]. Animal studies have demonstrated that opioids can accumulate in the developing brains of neonates, leading to delayed and disrupted brain tissue growth [15], [19], [20] These effects are similar to those observed in humans, where maternal morphine use during pregnancy has been associated with an increased risk of intrauterine fetal death, neonatal death, and growth retardation [21], [22].

Neuregulin-1 (NRG-1), a structural epidermal growth factor (EGF), plays a pivotal role in neuronal development [23], [24]. It contributes to neuronal differentiation, synaptic plasticity, and synaptogenesis [23], [24]. NRGs are transported through axons and presynaptic terminals, cooperating with ErbB receptors in what is known as the NRG-ErbB pathway [23], [24]. This pathway is essential for the formation of synapses and neuromuscular junctions, contributing to the overall maturation of the nervous system [23], [24], [25]. Alterations in NRG-ErbB activity influence various aspects of neuronal function and have been linked to neurological disorders, including autism spectrum disorders and schizophrenia [23], [24], [25].

Despite limited research on the effects of prenatal morphine exposure on offspring, its potential neurodevelopmental impact remains a significant concern. To address this gap, we conducted a study using a rat model to investigate the effects of prenatal morphine exposure on key neurodevelopmental processes. Specifically, we examined the expression of NRG-1 and ErbB4 genes in offspring, known to play critical roles in neurodevelopment, alongside inflammatory markers, oxidative stress markers, and BDNF levels in both maternal and neonatal blood and brain tissues. This comprehensive approach aims to provide insights into the potential mechanisms underlying the neurodevelopmental effects of prenatal morphine exposure.

## 2 Materials and methods

### 2.1 Animals

Twenty virgin female Wistar rats, aged 8 weeks and weighing 200–220 g, were obtained from the Laboratory Animal Research Center of Zahedan University of Medical Sciences (ZAUMS). The rats underwent a one-week acclimatization period in controlled conditions with a 12-hour light-dark cycle, a temperature of 22 ± 2 ˚C, and ad libitum access to food and water before the experiment. The study protocol received approval from the ethics committee for Animal Research at Zahedan University of Medical Sciences (ethical code: IR.ZAUMS.REC.1394.295).

### 2.2 Experimental design

**Fig. 1:**
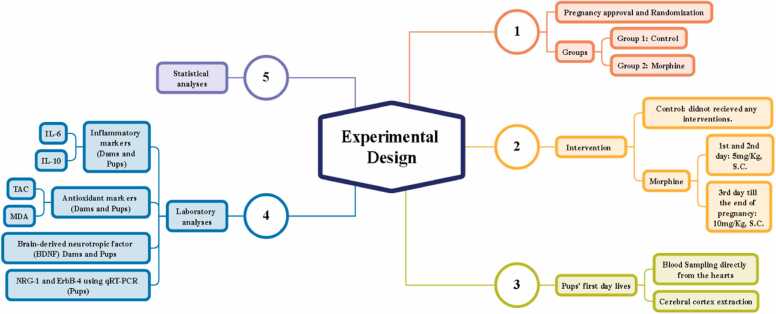
Experimental design overview.

As displayed in Fig. 1, for pregnancy induction, two female rats were housed with one male Wistar rat in a single cage. Each morning, the vaginal area of the females was examined for the presence of sperm. Upon detecting a positive sperm test, the impregnated females were isolated and maintained in separate cages [26], [27]. Following pregnancy induction, the female rats were randomly assigned to two experimental groups:

1. Control group: Animals in this group did not receive any interventions or treatments during pregnancy.
2. Morphine group: Animals in this group received morphine administration throughout the duration of pregnancy.

Animals assigned to the morphine group received morphine sulfate (Darou Pakhsh, CO, Tehran, Iran) injections according to the following schedule:

First and Second Days of Gestation: 5 mg/kg body weight administered subcutaneously (S.C.) daily. Third Day of Gestation Onward (until labor): 10 mg/kg body weight administered subcutaneously (S.C.) daily [28], [29], [30]. At the end of the gestational period (on day 21), both dams and naturally born pups were anesthetized using a combination of ketamine (100 mg/kg) and xylazine (10 mg/kg). Subsequently, blood samples were collected from the hearts of both dams and pups. The blood samples were centrifuged at 3500 rpm for 5 minutes to separate the serum. The serum samples were then stored at −70°C until they were analyzed for various parameters.

Brain cerebral cortex samples were collected from both the dam and her pups [31]. The samples were homogenized in phosphate-buffered saline (PBS) using a homogenizer. The homogenized samples were then centrifuged at 3500 rpm for 15 minutes. The supernatant was collected and stored at −70°C until analysis. Enzyme-linked immunosorbent assays (ELISAs) were performed using Zellbio (Germany) kits to determine the concentrations of interleukin-6 (IL-6), interleukin-10 (IL-10), and brain-derived neurotrophic factor (BDNF) in the brain cortex samples.

Additionally, calorimetric assay kits from Zellbio (Germany) were used to measure the levels of malondialdehyde (MDA), a marker of oxidative stress, and total antioxidant capacity (TAC) in the brain cortex samples.

### 2.3 Isolation of mRNA and analysis of quantitative real-time polymerase chain reaction (qRT-PCR)

Subsequent to the completion of pregnancy, the pups were utilized for molecular analysis. Following the dissection of the brain cortex from the pups under anesthesia (using Ketamine at 100 mg/kg and Xylazine at 10 mg/kg), the specimens were preserved at −80˚C.

To assess the gene expressions of NRG-1 and ErbB4, the brain tissue was homogenized, and mRNA extraction was carried out using the Total RNA Extraction Kit (Parstous, Iran). The purity and concentration of the extracted RNA were evaluated using the Thermo Scientific NanoDrop 2000 (ThermoFisher Scientific Inc, USA).

A cDNA Synthesis Kit (Parstous, Iran) was employed to perform reverse transcription of the total RNA into cDNA. Subsequently, a quantitative polymerase chain reaction (qPCR) protocol was executed, involving 1 cycle for 15 minutes at 95°C, followed by 45 cycles of 30 seconds at 95°C, 30 seconds at 61°C, and 30 seconds at 72°C. The real-time PCR was conducted using the CFX 96 real-time system (Bio-Rad, Bio-Rad laboratories Inc., California, United States). ß-actin was employed as the reference housekeeping gene for normalization.

Table 1 shows the employed primer sequences.

**Table 1:** Comparative Analysis of Morphine's Effects on Maternal Weight Gain, Litter Size, Birth Weight, and Brain Weight at Birth.

**Variable**	**Control**	**Morphine**	**p-Value**
**number of pups per litter**	10.20 ± 0.62	6.9 ± 0.43	= 0.0004
**Birth weight (gr)**	6.1 ± 0.076	5.8 ± 0.12	=0.0514
**Brain weight at birth (mg)**	261.6 ± 4.9	247.2 ± 6.03	=0.0806
**Maternal weight gain during pregnancy (g)**	146.3 ± 4.72	109.9 ± 3.99	<0.0001

### 2.4 Statistical analysis

The data were analyzed using GraphPad Prism 8 software and are presented as mean ± standard error of the mean (SEM). Statistical comparisons between groups for serum and brain tissue concentrations of IL-6, IL-10, BDNF, MDA, and TAC, as well as NRG1-ErbB4 gene expressions, were performed using the independent samples t-test. A p-value less than 0.05 was considered statistically significant.

## 3 Results

### 3.1 Morphine's Effects on Maternal Weight Gain, Litter Size, Birth Weight, and Brain Weight at Birth

As displayed in Table 1, the maternal weight gain during pregnancy was significantly lower in the morphine group (109.9 ± 3.99 g) compared to the control group (146.3 ± 4.72 g) (p-value <0.0001). The number of pups per litter was significantly lower in the morphine group (6.9 ± 0.43) compared to the control group (10.20 ± 0.62) (p-value = 0.0004). There was no significant difference in birth weight of pups between the control group (6.1 ± 0.076 g) and the morphine group (5.8 ± 0.12 g) (p-value = 0.0514). Similarly, there was no significant difference in brain weight at birth between the control group (261.6 ± 4.9 mg) and the morphine group (247.2 ± 6.03 mg) (p-value = 0.0806).

### 3.2 The effect of prenatal morphine exposure on NRG-1 and ErbB-4 gene expression levels

**Fig. 2:**
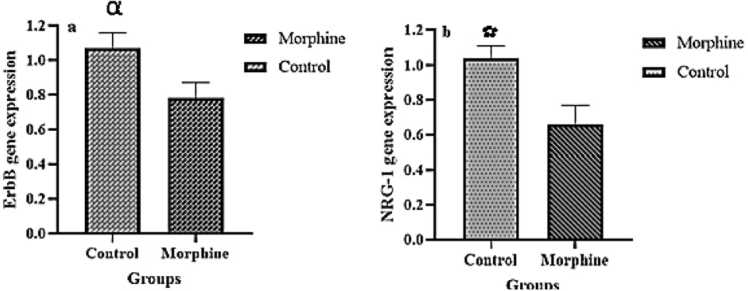
A: ErbB4 and NRG-1 expression levels reduced in morphine exposure rats in comparison with controls. A: ErbB4 gene expression, p=0.03; B: NRG-1 gene expression, p<0.01.

As shown in Fig. 2, the qrt-PCR analyses revealed a significant decrease in ErbB4 gene expression in morphine-exposed pups than in controls (0.78±0.28 versus 1.07±0.27, p=0.03). Moreover, the qrt-PCR results showed that NRG-1 expression levels in the morphine group were significantly lower than the control group (0.66±0.34 versus 1.04±0.21, p<0.01).

### 3.3 The effect of prenatal morphine exposure on IL-6 and IL-10 Concentrations

**Fig. 3:**
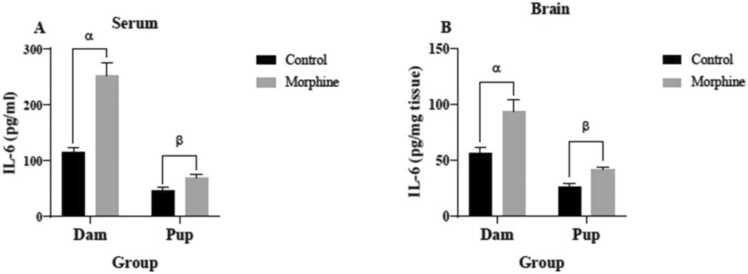
IL-6 concentrations in (A) serum and (B) brain tissue of control and morphine groups. A: α p<0/0001, morphine vs. control; β p<0.05, morphine vs. control. B: α p<0.01, morphine vs. control; β p<0.01, morphine vs. control.

As shown in Fig. 3A, the mean serum IL-6 levels in the dams (252.9±23.58 pg/ml) and pups (68.44±6.225 pg/ml) of the morphine group were significantly higher than the control group (115.3±7.831 pg/ml and 46.83±5.322 pg/ml, respectively) (p<0.0001 and p<0.05, respectively). Brain tissue IL-6 concentrations of the dams (94.13± 10.78 pg/mg-tissue) and pups (41.53± 3.077 pg/mg-tissue) of the morphine group were significantly higher than the control group (56.13± 5.802 pg/mg tissue and 26.72± 3.149 pg/mg-tissue) (both p<0.01) (Fig. 3B).

**Fig. 4:**
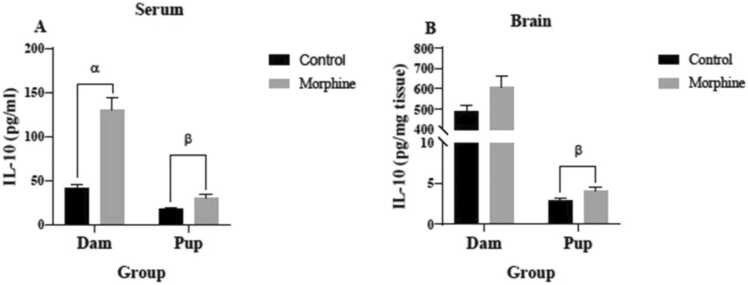
IL-10 concentrations in (A) serum and (B) brain tissue of control and morphine groups. A: α p< 0.0001, morphine vs. control; β p<0.05, morphine vs. control. B: β p<0.05, morphine vs. control.

As shown in Fig. 4A, serum IL-10 levels of the dams (130.2± 13.30 pg/ml) and pups (29.53± 4.552 pg/ml) of the morphine group were significantly higher than the control group (41.70± 4.006 pg/ml and 16.84± 2.082 pg/ml, respectively) (p<0.0001 and p<0.05, respectively). Mean IL-10 concentration of the pups’ brains (4.040± 0.4121 pg/mg-tissue) in the morphine group were significantly higher than the control group (2.868± 0.3344 pg/mg-tissue) (p<0.05) (Fig. 3B). IL-10 concentration in the dams’ brains (609.8± 51.80 pg/mg-tissue) of the morphine group were higher than the control group (486.3± 34.99 pg/mg-tissue), but this difference was not significant (p=0.06) (Fig. 4B).

### 3.4 The effect of prenatal morphine exposure on BDNF concentrations

**Fig. 5:**
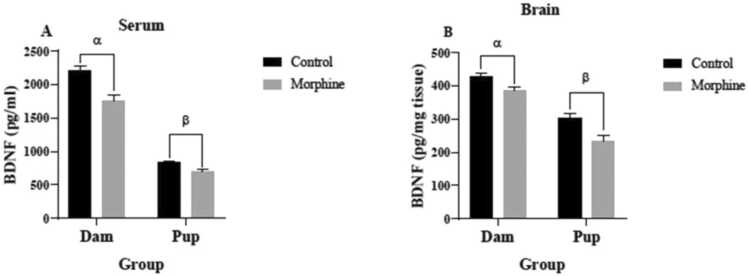
BDNF concentrations in (A) serum and (B) brain tissue of control and morphine groups. A: α p<0.001, morphine vs. control; β p<0.01, morphine vs. control. B: α p<0.01, morphine vs. control; β p<0.01, morphine vs. control.

Based on Fig. 5A, the mean serum BDNF levels of the dams (1755± 90.43 pg/ml) and pups (691.1± 32.71 pg/ml) of the morphine group were significantly lower than the control group (2212± 61.37 pg/ml and 830.8± 24.11 pg/ml, respectively) with p<0.001 and p<0.01, respectively. BDNF concentrations in brain tissues of the dams (388.3± 6.728 pg/mg-tissue) and the pups (234.8± 16.52 pg/mg-tissue) of the morphine group were significantly lower than the control group (427.5± 11.56 pg/mg- tissue and 304.3± 12.74 pg/mg-tissue, respectively) (both p<0.01) (Fig. 5B).

### 3.5 The effect of prenatal morphine exposure on TAC and MDA concentrations

**Fig. 6:**
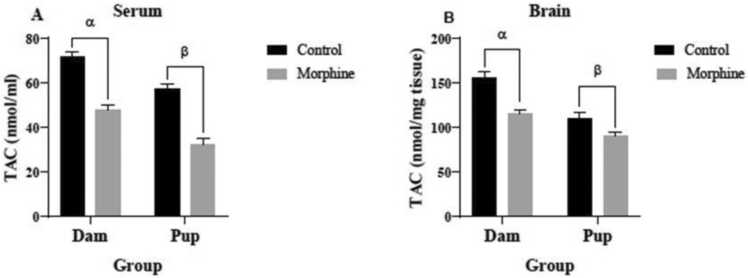
TAC level in (A) serum and (B) brain tissue of control and morphine groups. A: α p<0.0001, morphine vs. control; β p<0.0001, morphine vs. control. B: α p<0.0001, morphine vs. control; β p<0.05, morphine vs. control.

Serum TAC levels of the dams (48.08± 2.065 nmol/ml) and pups (32.5± 2.809 nmol/ml) in the morphine group were significantly lower than the control group (71.58± 2.554 nmol/ml and 57.38± 2.283 nmol/ml, respectively) (both p<0.0001) (Fig. 6A). Mean TAC concentration in the dams’ brains (114.9 ± 4.738 nmol/mg-tissue) of the morphine group were significantly lower than the control group (156.4± 5.688 nmol/mg-tissue) (p<0.0001) (Fig. 5B). TAC concentrations in pups’ brains (89.88± 4.797 nmol/mg-tissue) of the morphine group were significantly lower than the control group (110.6± 6.15 nmol/mg-tissue) (p<0.05) (Fig. 6B).

**Fig. 7:**
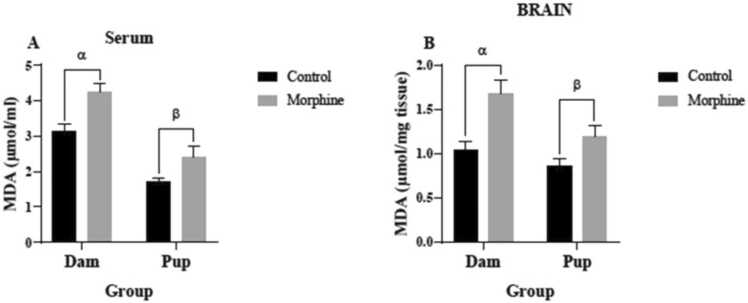
MDA level in (A) serum and (B) brain tissue of control and morphine groups. A: α p<0.01, morphine vs. control; β p<0.05, morphine vs. control. B: α p<0.01, morphine vs. control; β p<0.05, morphine vs. control.

Mean serum MDA levels of the dams (4.25± 0.2245 µmol/ml) and pups (2.406± 0.3008 µmol/ml) in the morphine group were significantly higher than the control group (3.125± 0.2004 µmol/ml and 1.717± 0.081 µmol/ml, respectively; with p<0.01 and p<0.05, respectively) (Fig. 7A). MDA concentrations in the brain tissues of the dams (1.675± 0.1562 µmol/mg-tissue) and pups (1.192± 0.1209 µmol/mg-tissue) of the morphine group were significantly higher than the control group (1.042 ± 0.09883 µmol/mg-tissue and 0.8667± 0.08469 µmol/mg-tissue, respectively; with p<0.01 and p<0.05 respectively) (Fig. 7B).

## 4 Discussions

In this research, we found that morphine has effects on the gene expressions of NRG1 and ErbB4 in the offspring brain. Additionally, we investigated the concentrations of IL-6, IL-10, BDNF, TAC, and MDA in both the offspring and mothers of rats administered with morphine. In summary, morphine led to decreased expressions of the NRG1-ErbB4 signaling pathway in the brain, reduced levels of BDNF and TAC, and increased levels of IL-6 IL-10, and MDA. These findings may provide insight into the underlying mechanisms of neurodevelopmental disorders associated with prenatal morphine exposure. NRG1, a multifaceted growth factor with diverse isoforms, plays a pivotal role in the development and functioning of the nervous system[25]. It exerts its effects through interactions with ErbB tyrosine kinase receptors, primarily ErbB4, influencing neurotransmission, glial cell generation, myelination, neuron-glial interactions, synaptogenesis, and neuronal migration during development [25], [32]. Our research revealed that the levels of NRG1 gene expression in the brain cortex of offspring from the morphine-exposed group were lower compared to the control group. Furthermore, we investigated the expression of the ErbB4 gene, which, similar to NRG1, showed significantly reduced levels in the offspring of the morphine-exposed group.

To our knowledge, no other studies have directly investigated the effects of morphine on NRG1 and ErbB4 expression in brain. However, Weingaertner et al. (2013) reported that chronic morphine treatment modulates the Neuregulin-ErbB signaling network, leading to reduced cell growth in human breast cancer cells [33].

Functionally, NRG1 expression, in the presence of cannabis, acts as an anxiolysis and is associated with Schizophrenia by disinhibiting the dopamine secreting substantia nigra neurons, effecting their adulthood firing rates [34], [35]. NRG1 expression is significantly reduced in the medial prefrontal cortex of rats that experienced prenatal asphyxia, without effecting the protein synthesis, suggesting long-lasting gene expression alterations [36]. Therefore, the causes of reduced NRG1 expressions like cannabis or morphine, or the adverse events that could lead to such reductions like asphyxia can have drastic and lasting effects on the physiology of certain parts of the brain as well as the molecular expression of the genes associated with those regions.

We found that dam and offspring exposure to morphine during pregnancy significantly increases inflammation, oxidative stress while reducing BDNF levels in both the brain and serum. Several studies have shown increased inflammation in morphine using pregnant mothers and their offspring. Raghavendra et al. (2002) found that chronic morphine administration increases spinal glial cells activities and pro-inflammatory cytokines such as IL-1, IL-6 and TNF-α in rats [37]. Stefania Merighi et al. (2012) showed that morphine exposure increases the secretion of pro-inflammatory cytokines such as TNF-α, IL-1, IL-6, and NO from microglial cells [38]. In humans, long-term morphine administration increases IL-6 levels in the cerebrospinal fluid [39].

The risk of neurological and psychiatric disorders increases in the offspring of mothers with greater levels of IL-6 during pregnancy [40], [41]. Rudolph et al. (2018) found that the mother’s inflammatory status during pregnancy can impact the development of her offspring’s brain [42]. They reported that higher levels of mothers’ IL-6 during pregnancy is associated with impaired neonatal neural circuits formation, leading to a reduced offspring working memory at two years of age [42]. Working memory is the ability to keep thoughts in mind, the impairment of which disrupts learning, mental and motor functions [42]. Graham et al. (2018) concluded that increased IL-6 levels in the mothers’ blood during pregnancy, impairs the fetal brain development (especially the amygdala structure) and may lead to behavioral changes associated with various psychiatric disorders [43]. The study by Rasmussen et al. (2019) indicated that increased IL-6 levels alongside systemic inflammation during pregnancy lead to changes in the fronto-limbic white matter tract of the brain leading to cognitive impairment early in life [44]. There is an inverse correlation between the concentration of IL-6 and the volume of the hippocampus [45], [46], [47]. Hippocampal defects cause cognitive, behavioral and psychologic disorders [45], [46].

Our results show that prenatal morphine exposure increases the release of inflammatory cytokines

(IL-6 and IL-10) in the brain cortexes of dams and their pups.

Previous studies have implicated the role of neuregulin-1 (NRG1) in the regulation of inflammation and immune responses. NRG1 is a growth factor that has been shown to reduce the release of pro-inflammatory cytokines in the brain and other tissues [48], [49].

Simmons et. al. (2016) indicated treatment with NRG1 inhibited IL-6 and TNF-alpha release after stroke in brain tissue of rats[48]. They suggested NRG1 has neuroprotective and anti-inflammatory effects that are associated with the differential regulation of NF-kB signaling pathways in microglia [48].

Lee et al. (2022) investigated the connection between NRG-1 and the alteration of neutrophil apoptosis by the regulation of cytokine release in human lung epithelial cells. They found that NRG-1 reduced the release of pro-inflammatory cytokines IL-6 and IL-8, suggesting that NRG-1 has anti-inflammatory effects.

Banerjee et al. (2022) assessed the role of NRG1 signaling in regulating cytokine and chemokine expression and secretion in granulosa cells [49]. They showed that knockdown of NRG1 in granulosa cells resulted in the enhanced expression and secretion of IL-6 and IL-10 [49].

Alizadeh et al. (2017) revealed that NRG1 treatment through intrathecal infusion attenuated the release of pro-inflammatory cytokines, TNF-α, and interleukin-1 beta (IL-1β) in acute spinal cord injury (SCI) while increasing the tissue levels of the anti-inflammatory cytokine, IL-10, in subacute SCI[50].

A human study by Marballi et al. (2010) showed that an NRG1 mutation was associated with schizophrenia [51]. They observed a significant increase in protein secretion levels of IL-6, TNF-α, and IL-8 in mutation carriers compared with controls [51].

In non-pregnant women, IL-10 acts as an immunosuppressant agent in response to inflammatory events [52]. IL-10 secretion occurs in many cells of the mother and the fetus during pregnancy [52] which suppress the maternal immune system, thus leading to the acceptance of the embryonic tissue and continuation of the normal pregnancy [52]. IL-10 provides part of its anti-inflammatory effects by reducing the secretion of inflammatory cytokines such as IL-6, IL-1, IL-12 and TNF-α [53].

Our study revealed elevated levels of the pro-inflammatory cytokine interleukin-6 (IL-6) in the cortices of both morphine-exposed dams and their offspring. This finding is consistent with previous studies showing that morphine exposure can increase IL-6 production [54]. Interestingly, we also found that prenatal morphine exposure led to increased levels of the anti-inflammatory cytokine IL-10 in the brain cortices of dams. The elevated IL-10 levels observed in the morphine groups in the present study may represent a compensatory mechanism to counteract the heightened inflammatory response induced by morphine exposure. IL-10, a key anti-inflammatory cytokine, is upregulated in response to inflammation, where its primary function is to suppress pro-inflammatory cytokines, including IL-6. This suggests that the elevated IL-10 levels in morphine-exposed groups could act as a feedback loop to dampen the inflammatory cascade triggered by morphine, potentially mitigating the adverse effects of morphine-induced inflammation. Previous studies have demonstrated IL-10's ability to inhibit IL-6 production, supporting its role in modulating the inflammatory milieu [55].

Our findings suggest that prenatal morphine exposure may alter NRG1 signaling pathways, potentially contributing to the observed effects on inflammatory responses. Given NRG1's established role in modulating inflammatory processes, these alterations could have significant implications for understanding the long-term effects of prenatal morphine exposure on brain development, particularly in the context of neurodevelopmental disorders. Chronic inflammation during critical developmental periods can disrupt normal brain processes, and persistent activation of immune pathways may contribute to neurodevelopmental disorders such as autism spectrum disorder (ASD), attention deficit hyperactivity disorder (ADHD), and schizophrenia [56], [57]. This highlights the potential significance of our findings for understanding the long-term impact of prenatal morphine exposure on brain development and the potential contribution of NRG1 signaling alterations to the pathogenesis of neurodevelopmental disorders.

Inflammation and oxidative stress have been implicated in the pathogenesis of neurodevelopmental disorders [58], [59], [60], and our findings suggest that prenatal morphine exposure may alter inflammatory responses in a way that contributes to the development of this disorder.

Our study demonstrates that morphine consumption in the pregnant dams induces oxidative stress in both the brain and serum of offspring. This aligns with previous findings in adult rodents showing that chronic morphine treatment reduces antioxidant enzyme activities, such as Catalase, Glutathione peroxidase, and Superoxide dismutase, and increases reactive oxygen species (ROS) and reactive nitrogen species (RNS) generation [61], [62], [63], [64], [65]. The observed oxidative stress during critical periods of brain development can have lasting effects on neuronal migration, synaptogenesis, and circuit formation, contributing to neurodevelopmental disorders such as ASD and intellectual disability [56], [57], [66]. Oxidative damage further contributes to neuroinflammation, excitotoxicity, and apoptosis [66], highlighting the potential link between prenatal morphine exposure, oxidative stress, and the pathogenesis of neurodevelopmental disorders.

BDNF is a member of the neuronal growth factors family and the major neurotrophin in the brain that induce neurogenesis, differentiation and survival of neurons [67], [68]. It also plays a role in the transcription and translation of proteins involved in the development of synapses, causing synaptogenesis and stability of synapses [69]. Kodomari et al. (2009) displayed that maternal BDNF levels in mice can cross the placenta into the fetal tissues and influence the fetal development [70]. Our findings demonstrate that morphine exposure led to decreased BDNF levels in both the serum and brain tissues of dams and their offspring. These observations align with previous studies reporting that morphine administration reduces BDNF levels in the brain and serum of rats, resulting in BDNF dysfunction within the central and peripheral nervous systems[71], [72]. Fanaei et al., (2020) demonstrated opium (morphine is one of the main constituents of opium.) consumption in pregnant women reduces BNDF levels in maternal and umbilical cord blood samples [73]. In addition, they showed that adverse pregnancy outcomes such as NICU admissions, congenital anomalies, neonatal deaths, meconium contaminated amniotic fluid, respiratory problems, neonatal resuscitation, and low Apgar scores, were significantly more common in the opium-addicted group than in the control group [73]. Ahmad Alipour et al. (2017) displayed that morphine injections during pregnancy decreased BDNF levels in the hippocampus of the female pups [74]. Han et al. (2008) showed that chronic morphine injections in rats reduced BDNF expression in the CA1 region of the hippocampus [75].

Research has established a close relationship between NRG1 and BDNF, highlighting their interactions during synaptogenesis [76]. NRG1, through the BDNF/TrkB signaling pathway, plays a critical role in regulating the survival and formation of synapses in immature primary cortical neurons [77]. Based on the findings of our study and those of previous research, it is plausible that morphine consumption during pregnancy disrupts NRG1 and BDNF signaling pathways. Our findings contribute to the understanding of the pathogenesis of neurodevelopmental disorders associated with prenatal morphine exposure. The observed alterations in NRG1/ErbB4 gene expression, coupled with changes in inflammatory markers and BDNF levels, suggest potential targets for therapeutic interventions. Further research is warranted to elucidate the precise mechanisms involved and explore potential therapies targeting the NRG1/ErbB4 pathway, which could mitigate the adverse effects of prenatal morphine exposure and lead to novel therapeutic strategies for neurodevelopmental disorders.

### 4.1 Limitations of the study

First, in our study, we administered morphine throughout pregnancy. However, prenatal morphine exposure during different stages of pregnancy may lead to varying effects on gene expression and behavior.

Second, our study used a single dose (10 mg/kg) of morphine. Dose-dependent effects might exist, and investigating a dose-response relationship could provide valuable insights.

## 5 Conclusion

Prenatal morphine exposure has been found to downregulate NRG1-ErbB4 expression in offspring brain, potentially related to a reduction in BDNF levels and an increase in inflammation and oxidative stress in the serum and brains of both dams and their offspring. These alterations may contribute to pregnancy complications and neurodevelopmental disorders observed in children born to morphine-consuming mothers. Further research is necessary to validate these suggestions and to delineate the specific molecular pathways involved.

## Ethics approval statement

This study was approved by Ethics Committee of Zahedan University of Medical Sciences (ethical code: IR.ZAUMS.REC.1394.295).

## Funding sources

Financial support for the study was conducted by the Office of Vice-Chancellor for Research and Information Technology of 10.13039/501100004847Zahedan University of Medical Sciences (code number: 7467).

## CRediT authorship contribution statement

**Samira Khayat:** Writing – original draft, Visualization, Validation, Methodology, Investigation, Formal analysis, Data curation, Conceptualization. **Hamed Fanaei:** Writing – original draft, Visualization, Validation, Supervision, Software, Resources, Project administration, Methodology, Investigation, Funding acquisition, Formal analysis, Data curation, Conceptualization. **Hamid Hafezinouri:** Project administration, Investigation. **Abdolhakim Ghanbarzehi:** Methodology, Investigation, Data curation. **Abolfazl Parsi-Moud:** Writing – review & editing, Visualization. **Ilia Mirzaei:** Writing – review & editing, Visualization, Supervision.

## Declaration of Competing Interest

The authors declare that they have no known competing financial interests or personal relationships that could have appeared to influence the work reported in this paper.

## Data Availability

Data will be made available on request.
